# *Desulfovibrio fairfieldensis*-Derived Outer Membrane Vesicles Damage Epithelial Barrier and Induce Inflammation and Pyroptosis in Macrophages

**DOI:** 10.3390/cells12010089

**Published:** 2022-12-25

**Authors:** Yawen Nie, Xiao-Qian Xie, Lingxi Zhou, Qijie Guan, Yilin Ren, Yong Mao, Jin-Song Shi, Zheng-Hong Xu, Yan Geng

**Affiliations:** 1 School of Life Sciences and Health Engineering, Jiangnan University, Wuxi 214122, China; 2 The Key Laboratory of Industrial Biotechnology, Ministry of Education, School of Biotechnology, Jiangnan University, Wuxi 214122, China; 3 National Engineering Laboratory for Cereal Fermentation Technology, Jiangnan University, Wuxi 214122, China; 4 Jiangsu Engineering Research Center for Bioactive Products Processing Technology, Jiangnan University, Wuxi 214122, China; 5 Department of Gastroenterology, Affiliated Hospital of Jiangnan University, Wuxi 214122, China; 6 Department of Oncology, Affiliated Hospital of Jiangnan University, Wuxi214122, China

**Keywords:** *Desulfovibrio fairfieldensis*, outer membrane vesicles, intestinal inflammation, pyroptosis

## Abstract

Sulfate-reducing bacteria *Desulfovibrio fairfieldensis* is an opportunistic pathogen that widely exists in the human intestine and can cause severe infectious diseases. However, the mechanisms contributing to its pathogenesis remain of great interest. In this study, we aim to investigate the outer membrane vesicles (OMVs) secreted by *D. fairfieldensis* and their pathogenic effect. The OMVs separated by ultracentrifugation were spherical and displayed a characteristic bilayer lipid structure observed by transmission electron microscopy, with an average hydrodynamic diameter of 75 nm measurement using the particle size analyzer. We identified 1496 and 916 proteins from *D. fairfieldensis* and its OMVs using label-free non-target quantitative proteomics, respectively. The 560 co-expressed proteins could participate in bacterial life activities by function prediction. The translocation protein TolB, which participates in OMVs biogenesis and transporting toxins was highly expressed in OMVs. The OMVs inhibited the expression of tight junction proteins OCCLUDIN and ZO-1 in human colonic epithelial cells (Caco-2). The OMVs decreased the cell viability of monocyte macrophages (THP-1-Mφ) and activated various inflammatory factors secretion, including interferon-γ (IFN-γ), tumor necrosis factor (TNF-α), and many interleukins. Further, we found the OMVs induced the expression of cleaved-gasdermin D, caspase-1, and c-IL-1β and caused pyroptosis in THP-1-Mφ cells. Taken together, these data reveal that the *D. fairfieldensis* OMVs can damage the intestinal epithelial barrier and activate intrinsic inflammation.

## 1. Introduction

The human gut contains a large number of microbes, which play an important role in human diseases and health. Sulfate-reducing bacteria (SRB) is a type of bacteria that exists widely in anaerobic environment, obtains energy by degrading organic matter, and reduces sulfate to sulfide. SRB is mainly composed of six categories in the human intestine, among which the most abundant species is *Desulfovibrio*. SRB is a major producer of hydrogen sulfide in the gut, and high concentrations of H_2_S in the gut can adversely affect the gut environment and gut microbiota through toxicity [1,2]. Studies have confirmed that SRB increase obviously in the intestine of patients with IBD and an increase in *Desulfovibrio* is particularly significant [3]. Among all *Desulfovibrio* species, *Desulfovibrio fairfieldensis* may be one of the most invasive and pathogenic strain that can cause severe bacteremia, appendicitis peritonitis, and choledocholithiasis [4,5,6].

We found that *D. fairfieldensis* can secrete outer membrane vesicles (OMVs) and pre-dicted that this macromolecule plays an important role in the occurrence and development of the disease. Outer membrane vesicles (OMVs) are double-layer lipid membrane nanospheres produced by Gram-negative bacteria, ranging in size from 20–400 nm [7]. OMVs encapsulate a variety of substances, including proteins, cell wall components such as lipooligosaccharides and peptidoglycans, nucleic acids, and metabolites [8]. Many recent studies have examined the links between OMVs and inflammation and found that OMVs could harm a variety of human cells and induce immune responses [9]. OMVs drive pro-inflammatory cytokine production by macrophages, which are specialized cells and play a defensive role when pathogens invade and participate in the repair of tissue damage. Stimulation of monocytes and macrophages with *Neisseria meningitidis* OMVs induced production of inflammatory factors such as interleukin-1β (IL-1β), interleukin-6 (IL-6), interleukin-10 (IL-10), and tumor necrosis factor alpha (TNF-α) [10]. Another study showed that *Porphyromonas gingivalis* did not activate inflammasome signaling involved in pyroptosis signaling in macrophages, but its secreted OMVs potently activated caspase-1 and produced large amounts of IL-1β and IL-18, which induced pyroptosis [11].

Studies have shown that OMVs can affect the tight junction of intestinal epithelial cells and cause impairment of intestinal epithelial barrier dysfunction. Intestinal epithelial barrier dysfunction usually refers to an increase in intestinal permeability, with accompanying features including loss of tight junction proteins between epithelial cells, translocation of the microbiome and their metabolites, and increased serum endotoxin and bacterial DNA [12]. Intestinal homeostasis relies on the complex and dynamic interactions between microbial flora, epithelial cells, and the host immune system. Bacteria may use OMVs to damage the gut barrier, enabling pathogenic components to enter the submucosa and promoting further pathological changes. The non-coding genetic materials carried by OMVs can interfere with normal communication between cells and disturb the homeostasis of the intestinal environment [13]. *Treponema denticola* OMVs could disrupt epithelial barrier function and substantially penetrate the cell layer [14]. *Campylobacter jejuni* OMVs had proteolytic activity and promoted bacterial invasion by mediating cleavage of E-cadherin and occludin in intestinal epithelial cells [15]. Therefore, the effect of OMVs on the intestinal epithelial barrier is one of the major ways that bacteria influence the host’s health.

In this study, we isolated *D. fairfieldensis* from the human gut and purified its OMVs. Next, we characterized *D. fairfieldensis* OMVs by transmission electron microscopy, dynamic light scattering, and proteomics. Then we studied the biological functions of OMVs in vitro. Human colonic epithelial cells (Caco-2) were used to study the effect of OMVs on the epithelial barrier function, and monocyte macrophages (THP-1-Mφ) were used to study the role of OMVs in inflammation and pyroptosis. THP-1 is a human-derived leukemia monocyte cell line, which is widely used in the research of human macrophages. THP-1 cells can be stimulated by phorbol ester (PMA) to differentiate into macrophages THP-1-Mφ [16]. Our results demonstrated that *D. fairfieldensis* OMVs could damage the epithelial barrier and activate inflammation and pyroptosis in macrophages. So far, no one has studied the *D. fairfieldensis* OMVs. We chose the *D.fairfieldensis* OMVs as the entry point to find its potential pathogenic effect. By revealing the possible impact of *D. fairfieldensis* on the host, basic research is done for the subsequent prevention and treatment of diseases caused by *Desulfovibrio*.

## 2. Materials and Methods

### 2.1. Bacteria Isolation

*Desulfovibrio* were isolated from healthy human feces. All samples were obtained with informed consent, and this study was approved by the Medical Ethics Committee of Wuxi Second People’s Hospital in accordance with the Declaration of Helsinki (No. 20170608). Bacteria were cultured in an enrichment culture medium (EDCM) [17] at 37 °C in an anaerobic incubator. The DNA was extracted, and the 16S rRNA gene was amplified and sequenced. Then the sequence was blasted against NCBI Ref Seq for taxonomic identification.

### 2.2. Purification and Characterization of Bacterial OMVs

OMVs were enriched by the ultracentrifugation method [18]. Briefly, *D. fairfieldensis* was cultured under anaerobic conditions at 37 °C until optical density (600 nm) reached 1.5. The bacteria-free supernatant was collected by centrifugation at 12,000× *g* for 15 min at 4 °C and then filtered through a 0.22 μm filter. OMVs were pelleted by ultracentrifugation at 200,000× *g* for 2 h at 4 °C in an ultracentrifuge [19] (Hitachi CP100WX, Tokyo, Japan). After removing the supernatant, OMVs were re-suspended in sterile PBS. We selected a 100 kDa (Merck Millipore, Billerica, MA, USA) ultrafiltration membrane to remove impurities such as flagella in the crude extract of OMVs, and then used a 50 kDa (Merck Millipore, Billerica, MA, USA) ultrafiltration membrane to enrich OMVs, and resuspended the retentate with sterile PBS [20]. The protein content of OMVs was assessed by the bicinchoninic acid protein assay kit (Thermo Fisher Scientific Inc., Waltham, MA, USA) according to the manufacturer’s instructions. The particle size of OMVs was detected by a particle size analyzer (Malvern, Worcestershire, UK).

### 2.3. Transmission Electron Microscopy (TEM)

OMVs were added to carbon coated copper mesh grid to hold for 1 min and stained with 1% uranyl acetate for 1 min. After the mesh grid dried, OMVs were imaged with the 80 kV Transmission electron microscope (Hitachi H-7650, Tokyo, Japan) [21].

### 2.4. Cell Culture

Caco-2 cells were cultured in Dulbecco’s modified Eagle’s medium (DMEM) (Thermo Fisher Scientific Inc, Waltham, MA, USA); THP-1 cells were cultured in RPMI 1640 medium (Thermo Fisher Scientific Inc, Waltham, MA, USA). All media were supplemented with 10% (vol/vol) heat-inactivated fetal bovine serum (FBS), penicillin (100 U/mL), and streptomycin (100 mg/mL) (Thermo Fisher Scientific Inc, Waltham, MA, USA). All cells were grown at 37 °C in an incubator with 5% CO_2_. Caco-2 cells were seeded at 4000 cells/well in a 96 well culture plate. THP-1 cells were induced with 120 nM PMA (CSNpharm, Chicago, IL, USA) for 72 h to differentiate into adherent THP-1-Mφ cells. For cell proliferation detection, THP-1-Mφ cells were seeded at 4000 cells/well in 96-well cell culture plates, treated with OMVs for 48 h, and then incubated with Cell Counting Kit-8 (TEYE Corporation, Shanghai, China) for 1–4 h at 37 °C. Absorbance at 450 nm was measured using a microplate reader.

### 2.5. Confocal Laser Imaging

THP-1-Mφ cells were cultured on a chamber slide, and the diluted green dye DiO (Beyotime Biotechnology, Shanghai, China) which can label the lipid structure with a final concentration of 5 μM was incubated with OMVs for 30 min at 37 °C. Subsequently, the excess dye was removed through centrifugation using a 50 kDa molecular weight ultrafiltration tube (Merck Millipore, Billerica, MA, USA). The labeled OMVs were resuspended in PBS, added to THP-1-Mφ cells, and incubated in the cell culture medium for 60 min. The diluted red fluorescent probe phalloidin (Beyotime Biotechnology, Shanghai, China) was used to label the cytoskeleton proteins in THP-1-Mφ cell membranes. After fixation with neutral formaldehyde, the THP-1-Mφ cell nuclei were labeled with DAPI (Beyotime Biotechnology, Shanghai, China).

### 2.6. Proteomics Analysis

*D. fairfieldensis* and their OMVs were subjected to label-free non-target quantitative proteomics analysis (BGI Genomics, Wuhan, China). The peptides separated by liquid phase chromatography were ionized by a nanoESI source and then passed to a tandem mass spectrometer Q-Exactive HF X for DDA (Data Dependent Acquisition) mode detection. The off-machine data were identified using the Andromeda engine integrated by MaxQuant, filtered at the spectrum level with PSM-level FDR <=1%, and at the protein level with protein-level FDR <=1% is further filtered. The final identified protein sequences were all from Uniprot-*Desulfovibrio* species number Database. Welch’s *t*-test was used for quantitative analysis of proteomics data. Fold change > 1.5 and *p* < 0.05 were used as the screening criteria for significantly different proteins.

### 2.7. Detection of Inflammatory Factors

After incubation with OMVs for 12 h, the cell culture supernatant of THP-1-Mφ cells was used to detect the secreted inflammatory factors using Bio-Plex Pro Human Cytokine Grp I Panel 27-plex Luminex liquid suspension chip through the Luminex 200 platform according to the manufacturer’s instruction (Luminex Corporation, Austin, TX, USA).

### 2.8. RNA Isolation and qRT-PCR

RNAs were extracted from cells using Trizol reagent (Invitrogen, Waltham, MA, USA) according to the manufacturer’s instructions and were reversely transcribed into cDNAs. The cDNAs were used for quantitative real-time PCR analysis using SYBR Green PCR Master Mix (Thermo Fisher Scientific Inc, Waltham, MA, USA). The following primers were used: *GAPDH*: forward: 5′-TGT GGG CAT CAA TGG ATT TGG-3′, reverse: 5′- ACA CCA TGT ATT CCG GGT CAAT-3′; *OCCLUDIN*: forward: 5′- ACA AGC GGT TTT ATC CAG AGT C-3′, reverse: 5′-GTC ATC CAC AGG CGA AGT TAA T-3′; *ZO-1*: forward: 5′- CAA CAT ACA GTG ACG CTT CAC A-3′, reverse: 5′- CAA CAT ACA GTG ACG CTT CAC A-3′; *ZO-2*: forward: 5′- ATG GAA GAG CTG ATA TGG GAA CA-3′, reverse: 5′- TGC TGA ACT GCA AAC GAA TGA A-3′.

### 2.9. Immunoblotting

Cells were lysed in RIPA buffer (Yeasen, Shanghai, China) and followed by 12% SDS-PAGE separation. Separated proteins were transferred onto polyvinylidene difluoride (PVDF) membranes (Merck Millipore, Billerica, MA, USA). The membranes were blocked by 5% bovine albumin in tris-buffered saline plus 0.1% Tween 20 for 1 h at room temperature. The membranes were probed with ZO-1 Antibody (PA5-21965, Thermo Fisher Scientific Inc., Waltham, MA, USA), OCCLUDIN Antibody (PA5-21965, Thermo Fisher Scientific Inc., Waltham, MA, USA), Gasdermin D Antibody (E8G3F, Cell Signaling, Danvers, MA, USA), Cleaved Gasdermin D Antibody (Asp275, Cell Signaling, Danvers, MA, USA), Caspase-1 Antibody (D7F10, Cell Signaling, Danvers, MA, USA, Danvers, MA, USA), IL-1β Antibody (D3U3E, Cell Signaling, Danvers, MA, USA), Cleaved-IL-1β Antibody (Asp116, Cell Signaling, Danvers, MA, USA) and then probed with anti-rabbit IgG Antibody (7074P2, Cell Signaling, Danvers, MA, USA). After washing with TBS-T, the membranes were visualized with SuperSignal West Pico PLUS substrate (Thermo Fisher Scientific Inc, Waltham, MA, USA). GAPDH Antibody (D16H11, Cell Signaling, Danvers, MA, USA) was used as a loading control.

### 2.10. Statistical Analysis

The data are represented by mean ± SEM. Student’s t test was used for analysis of differences between groups. *p* < 0.05 was considered significantly different. GraphPad Prism 8.2 (La Jolla, CA, USA) was used for statistical analysis and data visualization.

## 3. Results

### 3.1. Isolation and Identification of OMVs Secreted by D. fairfieldensis

**Figure 1:**
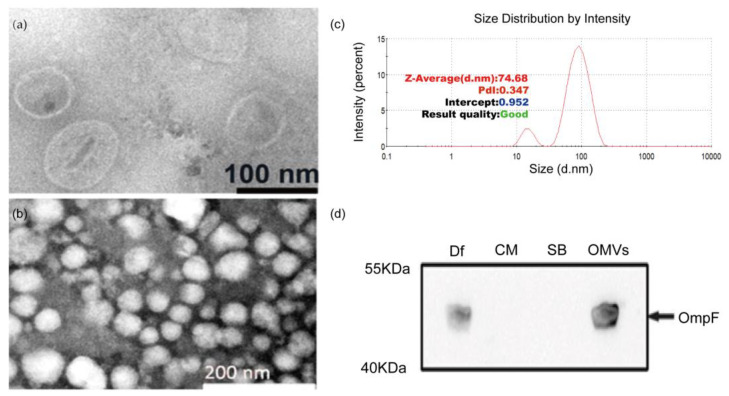
Morphology and membrane protein characterization of OMVs from *D. fairfieldensis*. (**a**,**b**) Negative-staining transmission electron microscopy of OMVs purified from *D. fairfieldensis*. (**c**) Nanoparticle-tracking analysis of OMVs was determined by Malvern particle size analyzer ZEN3700. (**d**) Western blot of specific OMV markers. Df: the abbreviation of *D. fairfieldensis*; CM: bacterial culture medium; SB: OMVs storage solution; OMVs: outer membrane vesicles.

We isolated *Desulfovibrio* from human feces with the enrichment medium, followed by molecular biological identification of the 16S rRNA gene. We obtained strains belonging to *D. intestinalis*, *D. simplex*, *D. legallii*, *D. Piger,* and *D. fairfieldensis*. Then, we measured the growth pH and H_2_S production ability of these strains. We found that the growth pH of *D. fairfieldensis* changed most obviously (Supplementary Table S1), and its H_2_S production ability was also the highest (Supplementary Table S2). Due to the association with gastrointestinal tract infections, *D. fairfieldensis* has been suggested to have more pathogenic potential than other *Desulfovibrio* [6]. We focused on *D. fairfieldensis* in the following experiment and we successfully purified OMVs from *D. fairfieldensis*. TEM showed that these OMVs were spherical and displayed a characteristic bilayer lipid structure (Figure 1a,b). The average hydrodynamic diameter of OMVs is 74.68 nm, as determined by the particle size analyzer (Figure 1c). The expression of OmpF, which is a positive marker of OMVs, confirmed that OMVs originated from blebbing of the outer membrane of *D. fairfieldensis* (Figure 1d).

### 3.2. Proteomic Characterization of D. fairfieldensis and Its OMVs

**Figure 2:**
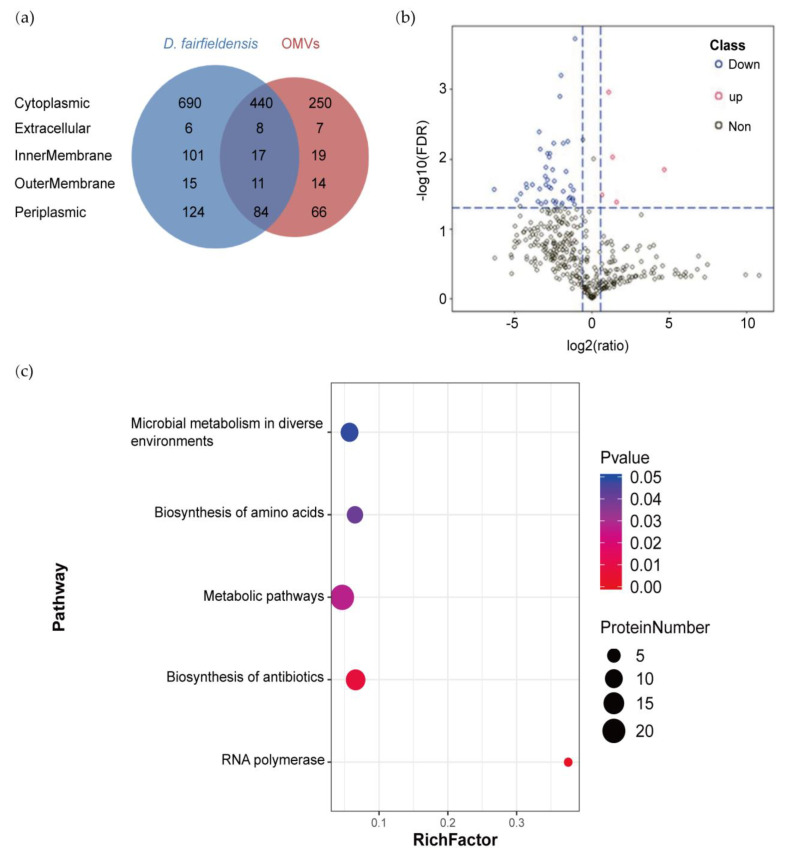
Proteomic analysis of *D. fairfieldensis* and its OMVs. (**a**) Venn diagram of proteins identified in OMVs and *D. fairfieldensis*. (**b**) Volcano plot of proteins expressed in *D. fairfieldensis* and its secreted OMVs. Red dots represent proteins significantly upregulated in OMVs, blues dots represent proteins significantly down-regulated in OMVs, and gray dots represent proteins without significant changes when compared with *D. fairfieldensis*. (**c**) Metabolic pathways enriched by differential proteins between OMVs and *D. fairfieldensis*. The *X*-axis enrichment factor (Rich Factor) is the number of differential proteins annotated to the pathway divided by all the proteins identified in the pathway. The larger the value, the greater the proportion of differential proteins to the pathway annotation. The dot size in the figure represents the number of differential proteins annotated to the pathway.

We identified 1496 and 916 proteins from *D. fairfieldensis* and its OMVs by HPLC-MS/MS analysis, respectively. Venn diagram displayed that 560 proteins were co-expressed in bacteria and its OMVs (Figure 2a). The proteins isolated from the parent bacteria and OMVs were predicted to be derived from cytoplasm, inner-membrane, periplasm, outer-membrane, and extracellular matrix. According to the recent hypothesis of extracellular microvesicle formation mechanism, it can be predicted that *D. fairfieldensis* can form OMVs by explosive lysis. A total of 50 differentially expressed proteins were identified (Figure 2b, Supplementary Table S3). Among them, five proteins were upregulated in the OMVs, including translocation protein TolB, which participates in OMVs biogenesis and transporting toxins (Figure 2b).

We then performed metabolic pathway analysis on the differential proteins between OMVs and *D. fairfieldensis*. The metabolic pathways enriched by these differential proteins have significant differences, including microbial metabolism involved in environmental changes (microbial metabolism in diverse environments), amino acid biosynthesis pathway (biosynthesis of amino acids), and antibiotic biosynthesis pathway (biosynthesis of antibiotics), metabolic pathways (metabolic pathways), and RNA-polymerase-related pathways (RNA polymerase) (Figure 2c).

**Figure 3:**
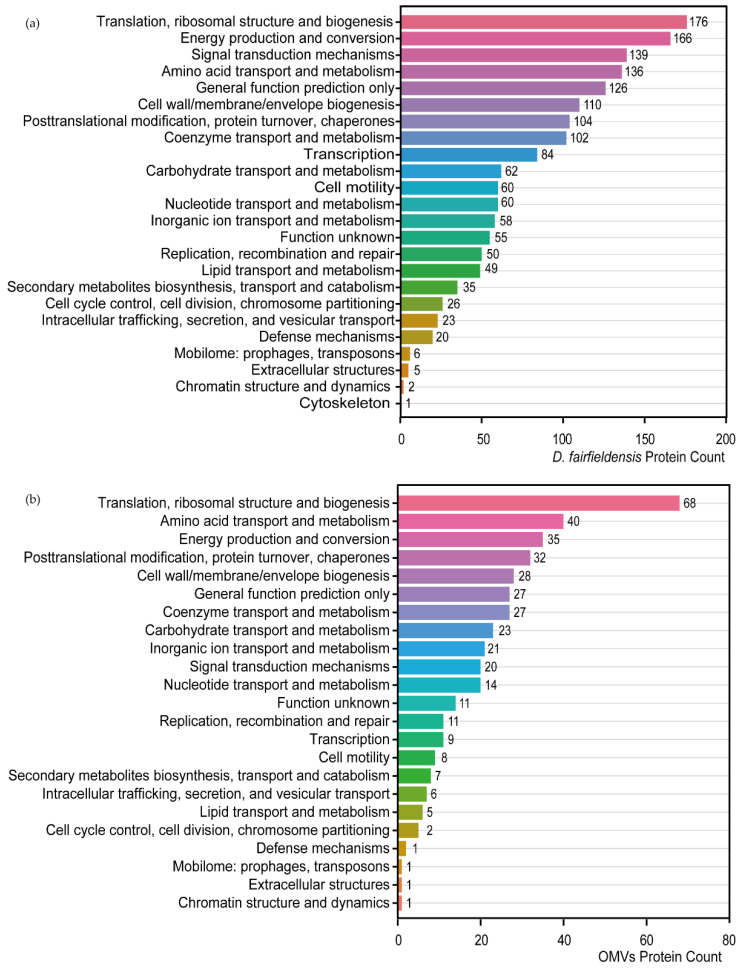
Protein function prediction of *D. fairfieldensis* (**a**) and its secreted OMVs (**b**) against COG database.

Proteins involved in translation, ribosomal structure, biogenesis, as well as energy production and conversion were present in the *D. fairfieldensis* and OMVs through protein function prediction (Figure 3a,b). Proteins involved in cytoskeleton category only exist in *D. fairfieldensis* (Figure 3a). In addition, proteins involved in the transport and metabolism of carbohydrates and coenzymes in the OMVs reflected that the OMVs could participate in and interfere with the life-related activities of the host (Figure 3b).

### 3.3. Disruption of the Tight Junction Structure of Intestinal Epithelium by D. fairfieldensis OMVs

**Figure 4:**
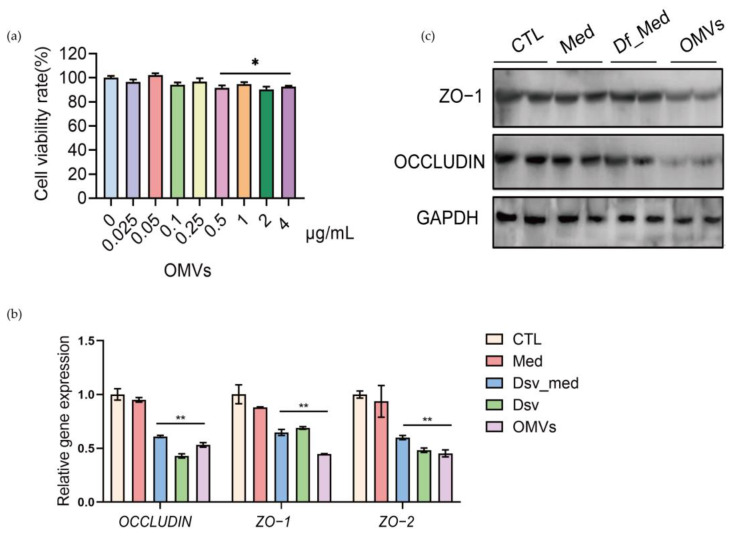
The effects of *D. fairfieldensis* and its secreted OMVs on Caco-2 cells. (**a**) The effects of OMVs on the proliferation of Caco-2 cells. (**b**,**c**) The effects of *D. fairfieldensis* and OMVs on Caco-2 tight junction proteins. CTL: Control; Med: Sterile *D. fairfieldensis* enrichment medium; Df_med: The culture supernatant of *D. fairfieldensis*; Dsv: *D. fairfieldensis*; OMVs: The outer membrane vesicles of *D. fairfieldensis*. * *p* < 0.05, ** *p* < 0.01 by Student’s *t* test in (**a**,**b**).

The tight junction proteins, including OCCLUDIN, ZO-1, and ZO-2, play an essential role in the gut barrier and mucosal repair [22,23]. We found that a higher amount of OMVs (0.5–4 μg/mL) significantly inhibited the proliferation of Caco-2 cells, but the cell viability rate was still above 80% (Figure 4a). We also found that the blank culture medium *D. fairfieldensis* (Med) did not impact the gene expression of tight junction proteins in Caco-2 cells (Figure 4b). Although the corresponding amount of the *D. fairfieldensis* culture supernatant (Df_Med) downregulated the relative gene expressions of ZO-1, ZO-2, and OCCLUDIN, its OMVs (1 μg/mL) exhibit a more substantial gene expression inhibition effect (Figure 4b). We further confirmed that OMVs markedly reduced the protein level of ZO-1 and OCCLUDIN in Caco-2 cells compared with other groups by Western blotting (Figure 4c). These data indicated that *D. fairfieldensis* OMVs impair the expression of tight junction proteins of the intestinal epithelium, which may facilitate their transfer to the host.

### 3.4. Phagocytosis of D. fairfieldensis OMVs and Stimulated Secretion of Inflammatory Factors by Human Mononuclear Macrophages

**Figure 5:**
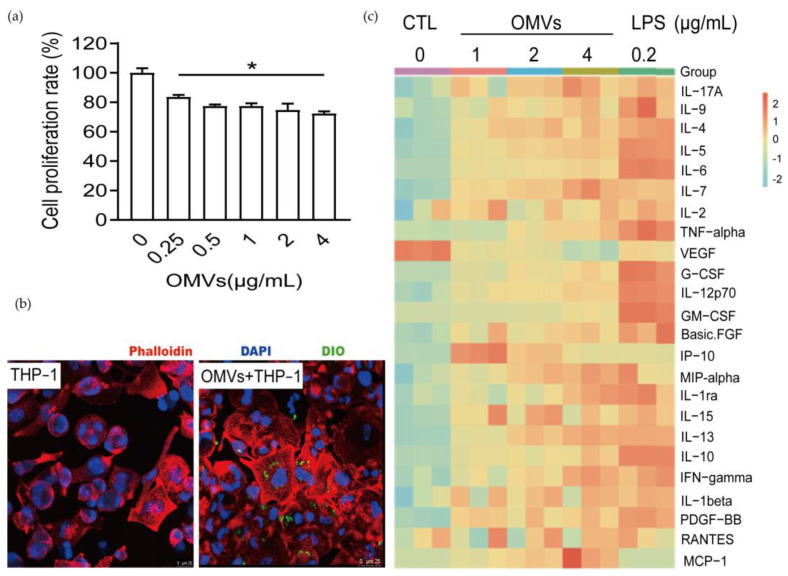
The effects of *D. fairfieldensis* OMVs on THP-1-Mφ cell. (**a**) Effects of OMVs on the viability of THP-1-Mφ cells. * *p* < 0.05 by Student’s *t* test. (**b**) The result of confocal laser tracing OMVs taken by THP-1-Mφ cells. Scale bar: 25 μm. The red fluorescent dye of phalloidin was used to label the cytoskeleton protein of THP-1 derived macrophages, the blue fluorescent dye of DAPI was used to label the nuclei, and the green fluorescent dye of DIO was used to label the lipid structure of OMVs. (**c**) Secretion of inflammatory factors by THP-1-Mφ cells stimulated by OMVs from *D. fairfieldensis*.

Macrophages can phagocytose antigens, so we speculate that *D. fairfieldensis* OMVs may be phagocytosed and cause inflammation. To investigate the effect of *D. fairfieldensis* OMVs on macrophages, we co-incubated the isolated OMVs with THP-1-Mφ cells. *D. fairfieldensis* OMVs significantly inhibited the proliferation of THP-1-Mφ cells in a dose-dependent manner (Figure 5a). After 30 min of co-incubation, THP-1-Mφ cells showed larger morphology, and the OMVs were inside the cells examined by the confocal laser scanning microscope (Figure 5b). A large amount of secreted inflammatory factors induced by the OMVs, including interlukin-17A (IL-7A), IL-1beta, IL-1ra, IL-2, IL-4, IL-5, IL-6, IL-7, IL-9, IL-10, IL-12p70, IL-13, IL-15, interferon-γ (IFN-γ), human macrophage inflammatory protein 1α (MIP-1α), and tumor necrosis factor (TNF-α) in THP-1-Mφ cells (Figure 5c). The cytokines including granulocyte colony stimulating factor (G-CSF), granulocyte-macrophage colony stimulating factor (GM-CSF), platelet derived growth factor (PDGF-BB), and basic fibroblast growth factors (FGF) were also up-regulated in THP-1-Mφ cells after the OMVs treatment (Figure 5c). The secretion of IFN-γ-induced protein 10 (IP-10) was much higher in lower dose than that of higher dose of the OMVs (Figure 5c). Both LPS and the OMVs decreased the secretion of vascular endothelial growth factor (VEGF) in THP-1-Mφ cells (Figure 5c). Co-incubation of THP-1-Mφ cells with LPS showed similar results except the chemokine monocyte chemokine-1 (MCP-1), which was only dose-dependently up-regulated in the OMVs group (Figure 5c). These, taken together, suggest that *D. fairfieldensis* OMVs can be phagocytosed by macrophages and activate cytokine secretion.

### 3.5. Pyroptosis of THP-1 Macrophages Caused by D. fairfieldensis OMVs

**Figure 6:**
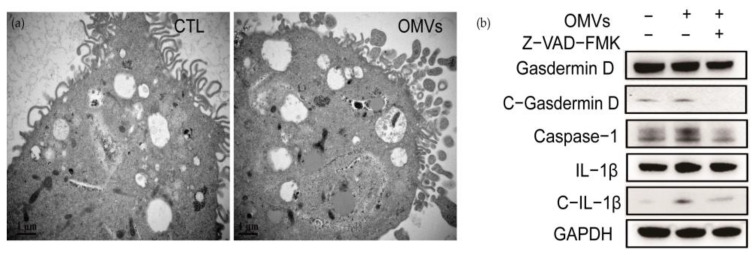
OMVs secreted by *D. fairfieldensis* caused THP-1-Mφ cell morphological changes and pyroptosis. (**a**) OMVs from *D. fairfieldensis* (1 μg/mL) stimulate pyroptosis of THP-1-Mφ cells. Scale bar: 1 μm. (**b**) Expression of inflammasome components by THP-1-Mφ cells induced by OMVs from *D. fairfieldensis* (1 μg/mL).

Pyroptosis is a regulated death pathway of cells [24,25]. Its main feature is that Gasdermin D protein is activated by caspase-1 cleavage to form peptides containing Gasdermin D N-terminal active domain, resulting in cell membrane perforation, cell rupture, and the release of contents, resulting in inflammation [26,27,28]. The TEM images of THP-1-Mφ cells stimulated by OMVs showed that the nuclear chromatin diffused and distributed around the nuclear membrane. At the same time, inflammasomes wrapped in multilayer membranes appeared. The microvilli of THP-1-Mφ cells were sparse, and vesicles were exuding in the cells, which was consistent with the characteristics of pyroptosis (Figure 6a). The OMVs induced the expression of the major inflammasome components, including cleaved-Gasdermin D, caspase-1, and c-IL-1β in THP-1-Mφ cells (Figure 6b). The addition of the caspase inhibitor Z-VAD-FMK can inhibit the cleavage of Gasdermin D and pro-IL-1β (Figure 6b). These results indicated that *D. fairfieldensis* OMVs could induce inflammatory cell death by activating the pyroptosis pathway.

## 4. Discussion

OMVs can participate in regulating the life activities of bacteria and can be used as a messenger of bacteria to communicate with the host [29,30]. In this study, *D. fairfieldensis* OMVs were identified and characterized. The functional analysis of the protein composition of OMVs revealed that its protein composition was roughly similar to that of the parent bacteria. *D. fairfieldensis* OMVs destroyed the tight junction barrier of intestinal epithelial cells. *D. fairfieldensis* OMVs also significantly induced the production of inflammatory factors and caused cell death of macrophages. These results provide a theoretical basis for further research on the physiological and pathological functions of the *D. fairfieldensis* OMVs.

The different protein profiles of *D. fairfieldensis* and its secreted OMVs indicate that they may have different biological functions. To confirm the secretion mode of OMVs, we located the proteins present in OMVs and found that they contained proteins in the outer membrane, inner membrane, periplasm, and cytoplasm. Therefore, we speculated that *D. fairfieldensis* produced OMVs through explosive lysis. In addition, we found that the proteins related to the carbohydrate and coenzyme transport and metabolism reflect to a certain extent that it participates in the function of bacteria and interferes with the life-related activities of the host. For example, the translocation protein TolB, which was upregulated in OMVs, plays an important role in the toxic effects of OMVs. TolB belongs to the Tol-Pal system, a highly conservative membrane system in Gram-negative bacteria, which is also an essential component of OMVs. The Tol-Pal system plays an important role in maintaining the stability and integrity of the cell membrane [31,32]. It is an essential device for pathogens to transport toxins and can also cause the host immune response [33]. Previous studies have shown that OMVs can promote bacterial colonization and regulate immune response [34,35]. The specific function of these OMVs may be related to the differential proteins in the executive function of OMVs. We found higher doses of OMVs lead to more cell death in THP-1-Mφ macrophages, either due to increased TolB proteins, or due to increased pyroptois. Whether TolB was the essential pathogenic agent in *D. fairfieldensis* OMVs needs further exploration.

As an important pathogenic agent secreted by Gram-negative bacteria, OMVs can easily penetrate the intestinal epithelium and even transport to a distant location in the host, thereby activating downstream cell signals and promoting the development of the disease. Enterohemorrhagic *Escherichia coli* can use outer membrane vesicles to target mitochondria, resulting in decreased mitochondrial transmembrane potential, cytochrome c transfer to cytosol, and apoptosis of intestinal epithelial cells [36]. OMVs secreted by intestinal microbes from colitis rats can down-regulate UGT1A1 expression in Caco-2 cells through a macrophage-mediated mechanism, thus causing intestinal ecological imbalance [37]. OMVs from *E. coli* BL21 significantly reduced the expression of the tight junction protein E-cadherin in Caco-2 and HT-29 cells, resulting in increased intestinal barrier permeability [38]. After co-culture of *D. fairfieldensis* OMVs with Caco-2 cells, we found that OMVs inhibited the proliferation of Caco-2 cells and decreased the expression of tight junction proteins in intestinal epithelial cells. Damage to the integrity of the intestinal barrier may result in the infiltration of *D. fairfieldensis* OMVs into the systemic circulation.

*E.coli* BL21-derived OMVs induced scorch death of a variety of cells, including bone marrow-derived dendritic cells, THP-1 macrophages, and HeLa cells [39]. Our research demonstrated that THP-1-Mφ macrophages could phagocytose *D. fairfieldensis* OMVs, which induced pyroptosis. OMVs can carry a variety of proteins, LPS, DNA fragments, and other pathogen-related molecules, which may cause inflammation by activating different signaling pathways [40,41]. Studies have shown that OMVs play an important role in bacterial survival and the spread of virulence to the host. However, we still need more research to reveal the pathogenic mechanisms of *D. fairfieldensis* and its secreted OMVs. Our study lacks the in vivo experimental part and does not explore the role of OMVs in the complex environment in vivo. Although the toxicity of OMVs has been demonstrated by in vitro cell experiments, it still needs to perform in vivo experiments to investigate their toxic dose and mechanism of action.

## Data Availability

The datasets generated and analyzed during the current study are available from the corresponding author on reasonable request.

## Supplementary Materials

The following supporting information can be downloaded at: https://www.mdpi.com/article/10.3390/cells12010089/s1. Table S1: Changes of pH of the culture system of *Desulfovibrio* species over time. Table S2: The content of H_2_S in the culture medium of *Desulfovibrio* species after 5 days of culture. Table S3: Differential proteins of *D. fairfieldensis* and its OMVs.
