# Supplementary material for: Desulfovibrio fairfieldensis-Derived Outer Membrane Vesicles Damage Epithelial Barrier and Induce Inflammation and Pyroptosis in Macrophages
Source: Cells. 2022 Dec 25;12(1):89. doi: 10.3390/cells12010089 (PMC9818291; doi:10.3390/cells12010089)
Supplement: Supplementary file 1 [file cells-12-00089-s001.zip › cells-2086755-supplementary.pdf]

cells MDPI

Supplementary Table S1. Changes of pH of the culture system of *Desulfovibrio* species over time.

Bacterial strain	pH			
	0d	2d	4d	6d
Desulfovibrio fairfieldensis	7.8	8.26	8.78	8.77
Desulfovibrio legallii	7.8	8.03	8.15	8.15
Desulfovibrio intestinalis	7.8	8.12	8.32	8.32
Desulfovibrio simplex	7.8	8.09	8.23	8.22
Desulfovibrio Piger	7.8	8.28	8.70	8.72

Supplementary Table S2. The content of H_2S in the culture medium of *Desulfovibrio* species after 5 days of culture.

Bacterial strain	H_2S (nmol/ml)
Desulfovibrio fairfieldensis	1101.99±51.20
Desulfovibrio legallii	637.31±15.06
Desulfovibrio intestinalis	801.45±24.57
Desulfovibrio simplex	713.96 ± 1.80
Desulfovibrio Piger	950.14±42.37

Supplementary Table S3. Differential proteins of *D. fairfieldensis* and its OMVs

Bacterial strain	H_2S (nmol/ml)	adj_pvalue
Serine--tRNA ligase OS	Down	0.001261
Molecular chaperone DnaJ OS	Down	0.00402
Hydroxylamine reductase OS	Down	0.007272
(Fe-S)-binding protein OS	Down	0.008358
Phosphomethylpyrimidine synthase OS	Down	0.008312
50S ribosomal protein L10 OS	Down	0.00594
Uncharacterized protein OS	Down	0.013163
Indolepyruvate oxidoreductase subunit IorA OS	Down	0.022833
Protein HflC OS	Down	0.017456
Uncharacterized protein OS	Down	0.014202
Universal stress protein OS	Down	0.023023

Phosphoribosylaminoimidazole-succinocarboxamide synthase OS	Down	0.027267
Dihydropyrimidine dehydrogenase OS	Down	0.009249
Isocitrate dehydrogenase (NADP(+)) OS	Down	0.026326
30S ribosomal protein S1 OS	Down	0.023247
Argininosuccinate lyase OS	Down	0.005521
Trigger factor OS	Down	0.025481
Uncharacterized protein OS	Down	0.00063
UTP--glucose-1-phosphate uridylyltransferase OS	Down	0.026579
50S ribosomal protein L7/L12 OS	Down	0.031478
Chaperone protein HtpG OS	Down	0.025362
Alpha-amylase OS	Down	0.01968
Enolase OS	Down	0.038087
DEAD/DEAH box helicase OS	Down	0.039645
Aminotran_1_2 domain-containing protein OS	Down	0.041221
Thiazole synthase OS	Down	0.04355
Hydrogenase OS	Down	0.040848
ATP synthase gamma chain OS	Down	0.047749
AFP-like domain-containing protein OS	Down	0.044597
DNA-directed RNA polymerase subunit beta OS	Down	0.043016

Phosphoribosylformylglycinamidine synthase subunit PurL OS	Down	0.040458
3-phosphoshikimate 1-carboxyvinyltransferase OS	Down	0.014923
DNA-directed RNA polymerase subunit alpha OS	Down	0.028979
Phosphate acetyltransferase OS	Down	0.0208
Vi polysaccharide biosynthesis protein VipB/TviC OS	Down	0.000189
DNA gyrase subunit B OS	Down	0.042895
Uncharacterized protein OS	Down	0.045359
Homoserine dehydrogenase OS	Down	0.005916
Glyceraldehyde-3-phosphate dehydrogenase OS	Down	0.036624
Uncharacterized protein OS	Down	0.024056
Type III pantothenate kinase OS	Down	0.027828
Isoleucine--tRNA ligase OS	Down	0.039108
DNA-directed RNA polymerase subunit beta' OS	Down	0.005269
HU family DNA-binding protein OS	Down	0.036831
Uncharacterized protein OS	Down	0.035479
Translocation protein TolB OS	Up	0.009346
DNA helicase OS	Up	0.014216
2-amino-3,7-dideoxy-D-threo-hept-6-ulosonate synthase OS	Up	0.001085

Uncharacterized protein OS	Up	0.041453
GTPase Der OS	Up	0.03274
